# Identification of microbial taxa present in *Ctenocephalides felis* (cat flea) reveals widespread co-infection and associations with vector phylogeny

**DOI:** 10.1186/s13071-022-05487-1

**Published:** 2022-10-31

**Authors:** Charlotte Manvell, Hanna Berman, Benjamin Callahan, Edward Breitschwerdt, William Swain, Kelli Ferris, Ricardo Maggi, Erin Lashnits

**Affiliations:** 1 Intracellular Pathogens Research Laboratory, College of Veterinary Medicine, North Carolina State University, Raleigh, NC USA; 2 Department of Population Health and Pathobiology, College of Veterinary Medicine and Bioinformatics Research Center, North Carolina State University, Raleigh, NC USA; 3 Department of Clinical Sciences, College of Veterinary Medicine, North Carolina State University, Raleigh, NC USA; 4 School of Veterinary Medicine, One Health Institute, University of California, Davis, CA USA; 5 Department of Medical Sciences, School of Veterinary Medicine, University of Wisconsin-Madison, Madison, WI USA

**Keywords:** Flea microbiome, *Bartonella*, *Wolbachia*, *Rickettsia*, Flea phylogenetics, Flea diversity, DNA barcoding, 16S NGS

## Abstract

**Background:**

*Ctenocephalides felis*, the cat flea, is the most common ectoparasite of cats and dogs worldwide. As a cause of flea allergy dermatitis and a vector for two genera of zoonotic pathogens (*Bartonella* and *Rickettsia* spp.), the effect of the *C. felis* microbiome on pathogen transmission and vector survival is of substantial medical importance to both human and veterinary medicine. The aim of this study was to assay the pathogenic and commensal eubacterial microbial communities of individual *C. felis* from multiple geographic locations and analyze these findings by location, qPCR pathogen prevalence, and flea genetic diversity.

**Methods:**

16S Next Generation Sequencing (NGS) was utilized to sequence the microbiome of fleas collected from free-roaming cats, and the *cox1* gene was used for flea phylogenetic analysis. NGS data were analyzed for 168 individual fleas from seven locations within the US and UK. Given inconsistency in the genera historically reported to constitute the *C. felis* microbiome, we utilized the decontam prevalence method followed by literature review to separate contaminants from true microbiome members.

**Results:**

NGS identified a single dominant and cosmopolitan amplicon sequence variant (ASV) from *Rickettsia* and *Wolbachia* while identifying one dominant *Bartonella clarridgeiae* and one dominant *Bartonella henselae/Bartonella koehlerae* ASV. Multiple less common ASVs from these genera were detected within restricted geographical ranges. Co-detection of two or more genera (*Bartonella, Rickettsia*, and/or *Wolbachia)* or multiple ASVs from a single genus in a single flea was common. *Achromobacter*, *Peptoniphilus*, and *Rhodococcus* were identified as additional candidate members of the *C. felis* microbiome on the basis of decontam analysis and literature review. *Ctenocephalides felis* phylogenetic diversity as assessed by the *cox1* gene fell within currently characterized clades while identifying seven novel haplotypes. NGS sensitivity and specificity for *Bartonella* and *Rickettsia* spp. DNA detection were compared to targeted qPCR.

**Conclusions:**

Our findings confirm the widespread coinfection of fleas with multiple bacterial genera and strains, proposing three additional microbiome members. The presence of minor *Bartonella, Rickettsia*, and *Wolbachia* ASVs was found to vary by location and flea haplotype. These findings have important implications for flea-borne pathogen transmission and control.

**Graphical Abstract:**

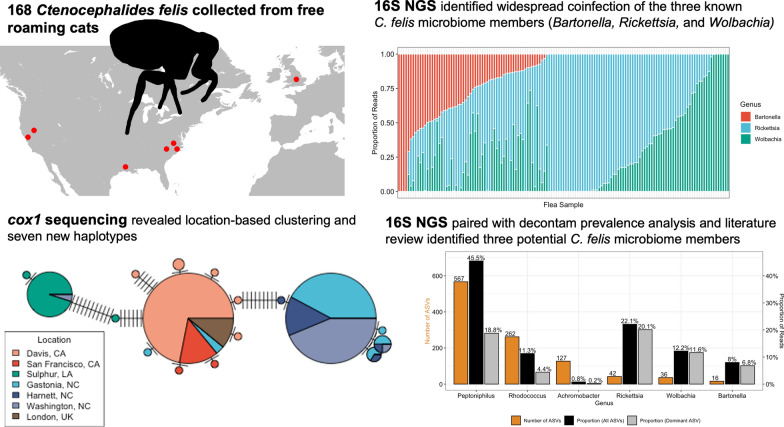

**Supplementary Information:**

The online version contains supplementary material available at 10.1186/s13071-022-05487-1.

## Background

Responsible for the transmission of multiple zoonotic pathogens, *Ctenocephalides felis*, the cat flea, is the most common ectoparasite of cats and dogs worldwide [1]. Despite this standing, few studies have attempted to characterize the microbiome of *C. felis*. Previous work has identified *Bartonella, Rickettsia*, and *Wolbachia* spp. as the dominant genera within the *C. felis* microbiome, but a more complete understanding of the flea microbiome including important questions related to coinfection of fleas by multiple pathogens and the relationship between microbial diversity and flea phylogeny remain poorly understood [2, 3].

The genera *Bartonella* and *Rickettsia* include multiple pathogenic species vectored by *C. felis* [4–6], with a broad range of zoonotic disease presentations that vary in severity [7–10]. Flea-associated *Rickettsia* spp. utilize *C. felis* as a reservoir host via vertical transmission [11, 12], whereas the *C. felis*-associated *Bartonella* spp. utilize the cat as a reservoir host, with free-roaming domestic cats (FRDC) serving as a source of maintenance within the environment [13, 14]. *Bartonella* spp. display geographic patterns consistent with *C. felis* [15, 16]. The diversity of *C. felis*-associated *Rickettsia* spp. has only recently been appreciated; therefore, little is known regarding their geographic distribution [17–20]. In addition to pathogenic *Bartonella* and *Rickettsia* spp., *Wolbachia* is widely accepted as a *C. felis* microbial constituent. *Wolbachia* spp. are closely related to *Rickettsia* spp. and are estimated to infect 66% of known insect species [21, 22]*. Wolbachia* spp. can exert a variety of effects on their hosts including manipulating host reproduction and altering pathogen transmission [23–25]. Research describing the *C. felis*-*Wolbachia* relationship, including the exchange of metabolites, epidemiology, and diversity of *Wolbachia* infecting *C. felis*, is in its infancy [26].

Beyond *Bartonella, Rickettsia*, and *Wolbachia*, research identifying additional members of the *C. felis* microbiome is difficult to interpret. As with other low biomass samples, sequencing-based methods for studying the *C. felis* microbiome suffers from the presence of contaminating DNA contributed from external sources, such as reagents or the laboratory environment [27, 28]. Contamination in low microbial biomass samples obscures community-wide measurements and complicates the identification of true microbiome members [29]. However, the careful use of control samples can help distinguish contaminants from microbes truly present in samples and thereby more accurately identify *C. felis* microbial genera [27, 28].

The ability of vector microbiome members to exert effects, whether positive or negative, on both the arthropod host and other microbes is well accepted. Because of the potential to influence pathogen transmission, describing these effects is a primary goal of many vector microbiome investigations [30]. The ability of specific members within the *C. felis* microbiome to alter the growth and reproduction of other microbes remains poorly described. A previous study of *Oropyslla* spp., a flea genus that primarily infests rodents, indicated a clear negative relationship between *Bartonella* and *Rickettsia* spp. [31]. This negative correlation between *Bartonella* and *Rickettsia* spp. has not been clearly demonstrated in *C. felis*, highlighting the need for further characterization of these genera within individual fleas. In ticks, infection with one *Rickettsia* spp. is known to interfere with transovarial transmission of a second *Rickettsia* spp. [32]. However, other reports indicate that coinfection with multiple *Rickettsia* species in ticks and fleas is possible. These coinfections are thought to result in decreased transmissibility, but the impact on transovarial transmission was not reported [33, 34]. Surveying the prevalence, distribution, and variability in *Bartonella, Rickettsia*, and *Wolbachia* spp. coinfections in individual fleas is critical to understand their interactions, with important implications for pathogen transmission.

In addition to the relationship between bacterial species carried by vectors, studies examining mosquitos and plant pathogen vectors document the importance of vector genotype on pathogen transmission dynamics [35, 36]. *Ctenocephalides felis* is a highly diverse species with four bioclimatically limited clusters originating from Africa [37]. Most published studies either have not determined the infection status of fleas surveyed solely for phylogenetic diversity or investigated only a small number of fleas from which both genotype and pathogen carriage was established [37–39].

The present study assessed the eubacterial diversity of individual *C. felis* collected from FRDC in diverse geographic locations via 16S rRNA NGS. The first aim was to use data on known pathogenic and commensal bacteria present in fleas (*Bartonella, Rickettsia*, and *Wolbachia* spp.) to remove contaminants and propose additional *C. felis* microbiome community members. To understand factors influencing pathogen presence in fleas, the second aim was to determine the presence and prevalence of each of these genera, including intra- and inter-genus coinfection, and compare known microbiome members to geographic location and vector phylogenetics. We expected to find location, flea genotype, and coinfection status variations among the three genera known to infect fleas, while identifying previously undescribed *C. felis* microbial genera. We also expected to find fleas colonized with multiple *Bartonella* and *Wolbachia* spp. ASVs, but only single *Rickettsia* spp. infections. Finally, the third aim was to compare NGS and qPCR for the detection of *Bartonella* and *Rickettsia* spp. in fleas, with the hypothesis that NGS would have lower sensitivity than qPCR for both pathogens.

## Methods

### Study design

This observational study examined fleas collected from FRDCs from six locations in the US and one in the UK: Davis, CA; San Francisco, CA; Sulphur, LA; Gastonia, NC; Harnett, NC; Washington, NC; London, UK. Fleas were collected from FRDCs when examined by veterinarians for spay or neuter through one local Trap-Neuter-Release (TNR) program in each location. All fleas were collected in March through July of 2019. The total numbers of fleas collected and cats sampled from each geographic location are displayed in Table 1.

**Table 1:** Number of cats and fleas sampled from each of the study locations

Location	Cats	Fleas
San Francisco, CA	19	11
Davis, CA	16	52
Sulphur, LA	6	21
Gastonia, NC	6	35
Harnett, NC	4	12
Washington, NC	5	30
London, UK	3	7

### Data and specimen collection

All cats presenting for TNR were combed for fleas regardless of demographic group or apparent ectoparasite presence. Fleas were then frozen prior to overnight shipment to the Intracellular Pathogens Research Laboratory at North Carolina State University. If more than six fleas were collected from a single cat, a random number generator in R was utilized to select six fleas for inclusion.

Fleas were visually identified to the species level with the assistance of Dr. James Flowers, Clinical Professor of Parasitology at the North Carolina State University College of Veterinary Medicine [40–42]. Individual fleas underwent two PBS and two ethanol washes. Washed fleas were then crushed by a magnetic bead beater until samples were fully homogenized. DNA was then extracted with the Qiagen DNeasy Blood and Tissue Kit (Qiagen, Valencia, CA, USA) following the manufacturer’s protocol for tissue extraction. Resulting DNA concentration (ng/μl) and purity (A_260_/A_280_) were determined spectrophotometrically (Nanodrop, Thermo Fisher Scientific, Waltham, MA, USA).

### Library preparation for 16S rRNA amplicon sequencing

Microbial community 16S rRNA gene amplicon sequencing was performed on DNA samples from individual fleas. DNA was submitted including 10 μl of DNA at 1.5 ng/μl per flea. Library preparation and sequencing were performed in July 2020 by the North Carolina State University Genomic Sciences Laboratory (Raleigh, NC) via the Illumina MiSeq system with primers targeting the 16S V3-V4 region. Sequencing relied on the forward primer 5ʹ-TCGTCGGCAGCGTCAGATGTGTATAAGAGACAGCCTACGGGNGGCWGCAG-3ʹ and the reverse primer 5ʹ-GTCTCGTGGGCTCGGAGATGTGTATAAGAGACAGGACTACHVGGGTATCTAATCC-3ʹ, selected from Klindworth et al. [43]. Ten negative extraction controls were included in sample processing and library preparation. These extraction controls were generated by performing manual DNA extraction of PBS at the same time as flea samples.

### Sequence preparation and filtering

Following library preparation, sequence data were prepared and analyzed in R (version 4.0.4) first utilizing DADA2 [44] version 1.20.0 to inspect quality profiles, filter, and trim sequences and then infer amplicon sequence variants (ASVs) and remove chimeras. Taxonomy was assigned using the DADA2 assignTaxonomy() function and the non-redundant Silva taxonomic training database version 138.1 (“silva_nr99_v138.1_train_set.fa”, https://www.arb-silva.de/). Species identity was assigned by the Silva species database version 138.1 (“silva_species_assignment_v138.1.fa.gz”, https://www.arb-silva.de/) based on exact matching.

Filtering was first performed to remove ASVs with fewer than three reads. Decontam [45] was then employed to identify potential members of the microbiome based on the scores of the three genera which are known *C. felis* microbiome constituents: *Bartonella, Rickettsia*, and *Wolbachia*. The decontam prevalence method is built on the premise that contaminant ASVs will be present more often in negative controls than they are in true samples. Decontam generates a score that discriminates between likely contaminants (scores closer to zero) and likely non-contaminants (scores closer to one). All genera with one or more ASV(s) above the score assigned to known microbiome members were considered potential microbiome members and reported. Literature review was utilized to select genera from this list to be considered candidate true microbiome members. To perform the literature review, all potential microbiome genera were searched on PubMed in combination with “microbiome,” “flea,” “tick,” and “mosquito” to identify relevant reports. The identified literature was examined on the location of detection and rigor of filtering to select only the genera which possess considerable evidence to be considered candidate microbiome members. Of those genera selected as candidate microbiome members, ASVs detected in more than three of the extraction control samples were removed.

### ASV analysis

Amplicon Sequence Variants (ASVs) are unique sequences generated by NGS after the correction of errors in amplicon sequencing data by the DADA2 method. Each ASV represents a group of organisms that share identical sequences over the amplified region of the 16S rRNA gene. Short-read 16S ASVs, such as those analyzed here, correspond roughly to species-breadth units, but can differentiate sub-species clades within some bacterial species. To compare the phylogenetic relationship between ASVs, the R package ape was utilized to build a neighbor-joining tree to compare the relatedness of ASVs [46, 47]. The Interactive Tree of Life (iTOL) software allowed tree visualization and annotation [48].

### Pathogen detection with qPCR

Quantitative polymerase chain reaction (qPCR) was performed for the amplification of *Anaplasma*, *Bartonella*, *Ehrlichia, Mycoplasma*, and *Rickettsia* spp. DNA. Table 2 lists the targeted genes used to amplify DNA of each pathogen. Each reaction included three controls: molecular-grade water non-template control, negative control from a known negative cat, and positive control plasmid. All positive samples were confirmed by sequencing (GENEWIZ Inc., Raleigh, NC). Sequence cleanup and viewing were performed in SnapGene (Insightful Science, San Diego, CA) and alignment with NCBI BLAST (http://blast.ncbi.nlm.nih.gov/Blast) [49]. Only fleas from which a sequence was acquired were considered positive by qPCR.

**Table 2:** Specific oligonucleotide names and primer sequence targets selected for pathogen qPCR amplification

Target organism	Oligonucleotide name	Oligonucleotide sequence (5′-3′)	Target gene	PCR product size (bp)	References
*Bartonella* spp.	Bart_ssrA_F	GCTATGGTAATAAATGGACAATGAAATAA	*ssrA*	158	[92]
	Bart_ssrA_R3	GACAACTATGCGGAAGCACGTC			
*Bartonella* spp.	BsppITS325s	CCTCAGATGATGATCCCAAGCCTTCTGGCG	ITS	130	[87]
	BsppITS543as	AATTGGTGGGCCTGGGAGGACTTG			
	BsppITS500p	FAM-GTTAGAGCGCGCGCTTGATAAG- IABkFQ			
*Rickettsia* spp.	Rick23-5_F2	AGCTCGATTGATTTACTTTGCTG	23S-5S	247	[92]
	Rick23-5_R	TTTGTATTGCTAGCTTGGTGG			
*R. felis*	RifelisOmpA-172 s	AGTCCTTGGTGCTGCAAGAACCGTAACTG	OmpA	160	This study
	RifelisOmpA-330as	ACCACTGAACCTAATGAAATATCACCAGT			
*R. asembonensis*	RiasemboOmpA-175 s	GTTGGGAGGAACAACGATAGATGCA	OmpA	120	This study
	RiasemboOmpA-245as	ACCGTAAATAAACCAGGAGCAAAACCA			
*Mycoplasma* spp.	Myco_Hf_F.1	GACGAAAGTCTGATGGAGCAAT	16S rRNA	127	[13]
	Myco_Hf_R	ACGCCCAATAAATCCGRATAAT			
*Anaplasma* and *Ehrlichia* spp.	AE16S_45F	AGCYTAACACATGCAAGTCGAACG	16S rRNA	199	[90]

The target gene, product size, and reference are listed for each organism

*Rickettsia* spp. detection was improved utilizing species-specific primers for *R. felis* and *Rickettsia asembonensis* (Table 2). Each qPCR reaction was performed with a 25 μl reaction volume consisting of 12.5 μl SsoAdvanced Unviersal SYBR Green Supermix 2X (Bio-Rad), 7.1 μl of molecular grade water, 5 μl DNA, and 0.2 μl of each primer. The reaction began with denaturation at 98 ℃ for 3 min, followed by 45 cycles of denaturation at 98 ℃ for 15 s, annealing at 66 ℃ for 15 s, and extension at 72 ℃ for 20 s, followed by a melt curve.

NGS sensitivity and specificity for the detection of *Bartonella* and *Rickettsia* spp. DNA were calculated utilizing qPCR as the reference standard.

### Flea phylogenetic analysis with cox1 cPCR

Flea phylogenetic group was assigned by sequencing of the cytochrome c oxidase subunit I (*cox1*) gene amplified by conventional PCR (cPCR) utilizing the Cff-F and Cff-R primers designed by Lawrence et al. [39]. The cPCR reaction was performed with a 25 μl reaction volume consisting of 12.5 μl 2 × MyTaq HS Red Mix (Meridian Bioscience, Cincinnati, OH), 11.1 μl molecular grade water, 1 μl DNA, and 0.2 μl each primer. The reaction began with denaturation at 95 ℃ for 2 min, followed by 40 cycles of denaturation at 95 ℃ for 30 s, annealing at 55℃ for 30 s, and extension at 72 ℃ for 30 s, ending with a final extension step at 72 ℃ for 10 min. Fleas from which a clean sequence was not acquired were retested at 0.01, 0.1, and 4 µl DNA per reaction to achieve successful amplification. DNA extracts from which *C. felis* DNA could not be amplified via *cox1* cPCR were excluded from analysis.

Haplotype networks were constructed based on a randomized minimum spanning tree, both of which were generated using the pegas package [50]. Specific visualizations of these haplotype networks were then developed to compare flea location and the occurrence of individual ASVs.

Analysis of flea phylogenetic sequences required the creation of a neighbor-joining tree. Detected haplotypes were compared to those reported by Lawrence et al. [37]. α-Diversity was analyzed via calculation of net relatedness index (NRI) [51]. This calculation is based on nodal distance with more negative communities being more phylogenetically diverse. The β-diversity measure PhyloSor was calculated to quantify the phylogenetic similarity between communities with more positive values indicating more genetically similar communities [52]. A reference *Ctenocephalides canis cox1* sequence (accession number MW136242.1) served as the outgroup for β-diversity calculations.

All analyses were performed in the R statistical computing environment utilizing the packages ape [46], Biostrings [53], dada2 [44], dplyr [54], decontam [45], filesstrings [55], GEOquery [56], ggforce [57], ggplot2 [58], ggsci [59], here [60], janitor [61], PhyloMeasures [62], phyloseq [63], picante [64], magrittr [65], RVAideMemoire [66], and vegan [67].

## Results

A total of 182 fleas were collected; 168 of these fleas passed *cox1* cPCR control. These fleas were collected from 75 cats with the number of fleas and cats from each geographic location reported in Table 1.

**Fig. 1:**
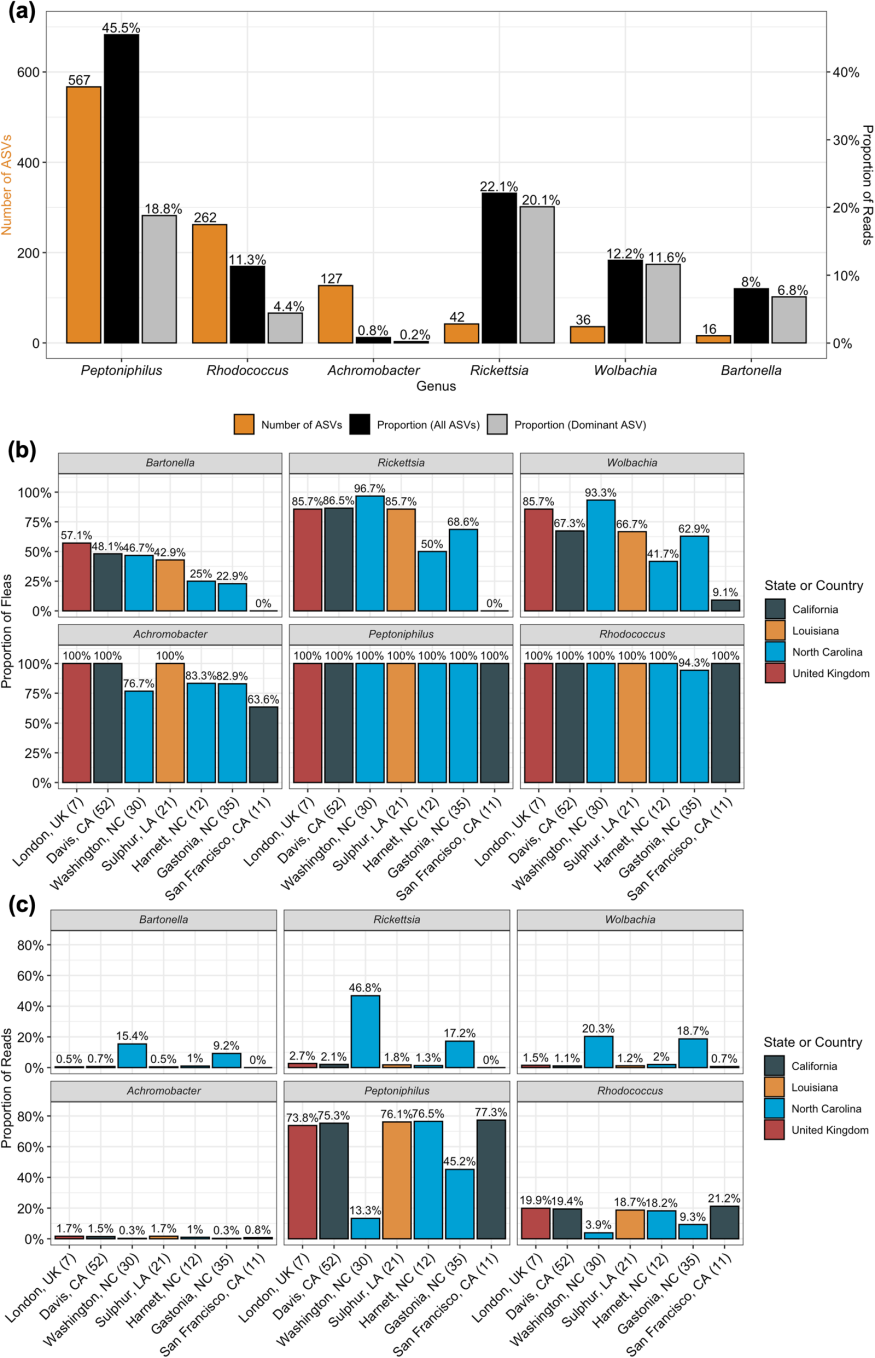
**a** Total number of ASVs (orange) assigned to each of the six genera indicated as most likely to be true microbiome members, as well as the proportion of reads assigned to each genera (black) and the proportion of reads assigned to the most abundant ASV of that genera (gray). **b** Proportion of fleas from each location with one or more read from the indicated genus. **c** Proportion of reads from each location assigned to each genus. The total number of fleas from each location was included in parentheses after the location on the x-axis

A total of 12,637 ASVs assigned to 457 genera were inferred by DADA2 across the flea and negative control samples; 1569 of these ASVs were removed as they were represented by fewer than three reads across all samples. *Peptoniphilus* ASVs were found in all 168 fleas, followed by *Rhodococcus* (167 fleas), *Achromobacter* (149 fleas), *Rickettsia* (128 fleas), *Wolbachia* (111 fleas), and *Bartonella* (63 fleas). This order was similar to that found when observing the total number of ASVs and proportion of reads for each genus (Fig. 1a). *Wolbachia, Rickettsia*, and *Bartonella*, but not *Achromobacter*, *Peptoniphilus*, and *Rhodococcus*, derived a majority of their reads from a single most abundant ASV (Fig. 1a).

The decontam prevalence method indicated that the most abundant *Wolbachia, Rickettsia*, and *Bartonella* ASVs were assigned a score of 0.97, 0.92, and 0.89, respectively. Therefore, we set a threshold of ≥ 0.89 to consider a genus as a potential microbiome member. Thirteen of 457 (3%) genera exceeded this threshold (Additional file 1: Fig. S1): *Achromobacter* [68, 69]*, Anaerococcus* [70–73]*, Bacteroides* [74, 75]*, Blastomonas* [76, 77], *Clostridium *sensu stricto* 5* [78], *Lachnoclostridium* [79]*, Methanothermobacter* [80]*, Peptoniphilus* [81, 82]*, Peptostreptococcus* [82, 83], and *Rhodococcus* [84, 85]. A literature search evaluating previous reports of these genera and the rigor of the contaminant filtering in these publications supported the idea that three of these genera (*Achromobacter*, *Peptoniphilus*, and *Rhodococcus*) should be considered candidate *C. felis* microbiome members.

*Anaplasma, Ehrlichia*, and hemotropic *Mycoplasma* DNA was not amplified from any of the fleas by either qPCR or NGS.

When examining the number of fleas with at least one read from *Achromobacter*, *Bartonella, Rickettsia*, or *Wolbachia*, there were geographic location-based differences in prevalence not observed in *Peptoniphilus* or *Rhodococcus* (Fig. 1b). There were also differences in the proportion of reads from each location assigned to the identified genera with Washington, NC, and Gastonia, NC, consistently reporting the highest proportion of *Bartonella, Rickettsia*, or *Wolbachia* reads (Fig. 1c).

**Fig. 2:**
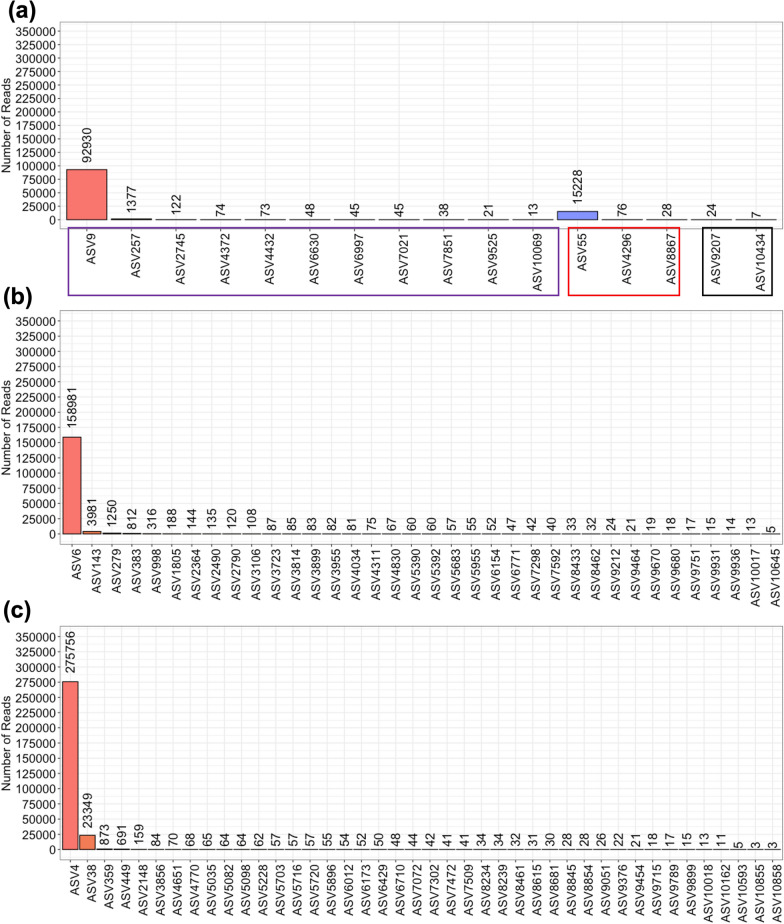
Number of total NGS reads assigned to each *Bartonella* (**A**), *Rickettsia* (**B**), and *Wolbachia* (**C**) ASV. *Bartonella* ASVs are divided by the *B. clarridgeiae*-like (purple), *Bh/Bk*-like ASVs (red), and other ASVs (black)

**Fig. 3:**
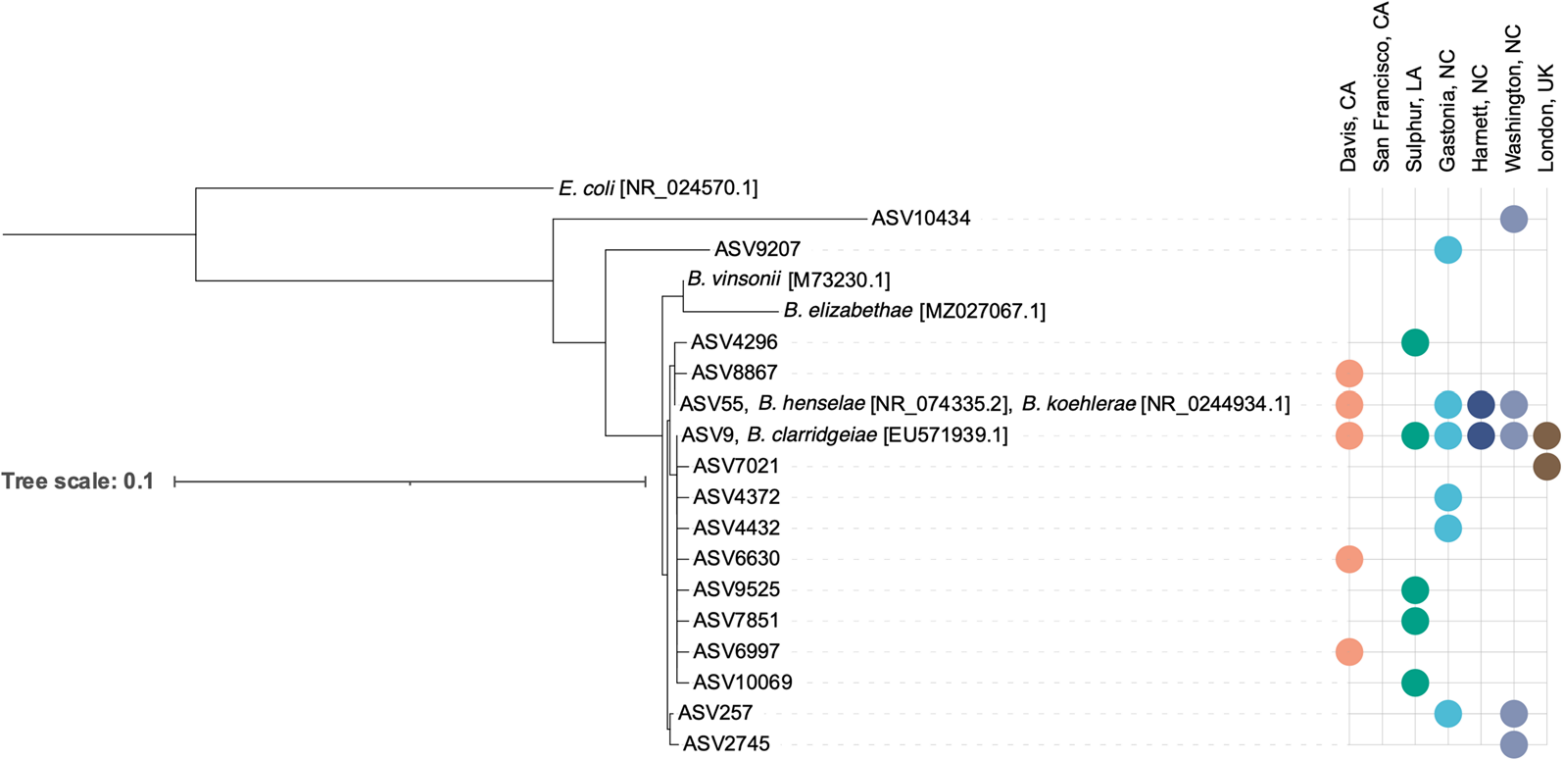
Phylogenetic tree displaying the homology of *Bartonella* ASVs compared to sequences of known origin with GenBank reference indicated in brackets. Colors in the dot plot indicate the location(s) at which specific ASVs were detected; the locations are labeled on the x-axis

NGS yielded 16 distinct *Bartonella* spp. ASVs (Fig. 2a). A majority of the *Bartonella* ASVs detected aligned closely with either *B. clarridgeiae* (*Bc*-like ASVs) or *B. henselae/B. koehlerae* (*Bh/Bk*-like ASVs) (Fig. 3). A single dominant ASV was found in both groups: ASV9 and ASV55. ASV9 was detected in all locations except for San Francisco, CA, and ASV55 was detected in all locations except for San Francisco, CA, Sulphur, LA, and London, UK. One of 21 fleas from Sulphur, LA, contained ASV4296, a *Bh/Bk*-like ASV. All minor *Bc*-like or *Bh/Bk*-like ASVs were found in a single location or two North Carolina locations. Two outlier *Bartonella* ASVs (ASV9207 and ASV10434) were detected in a single flea each. Neither of these ASVs aligned with known *Bartonella* spp.

**Fig. 4:**
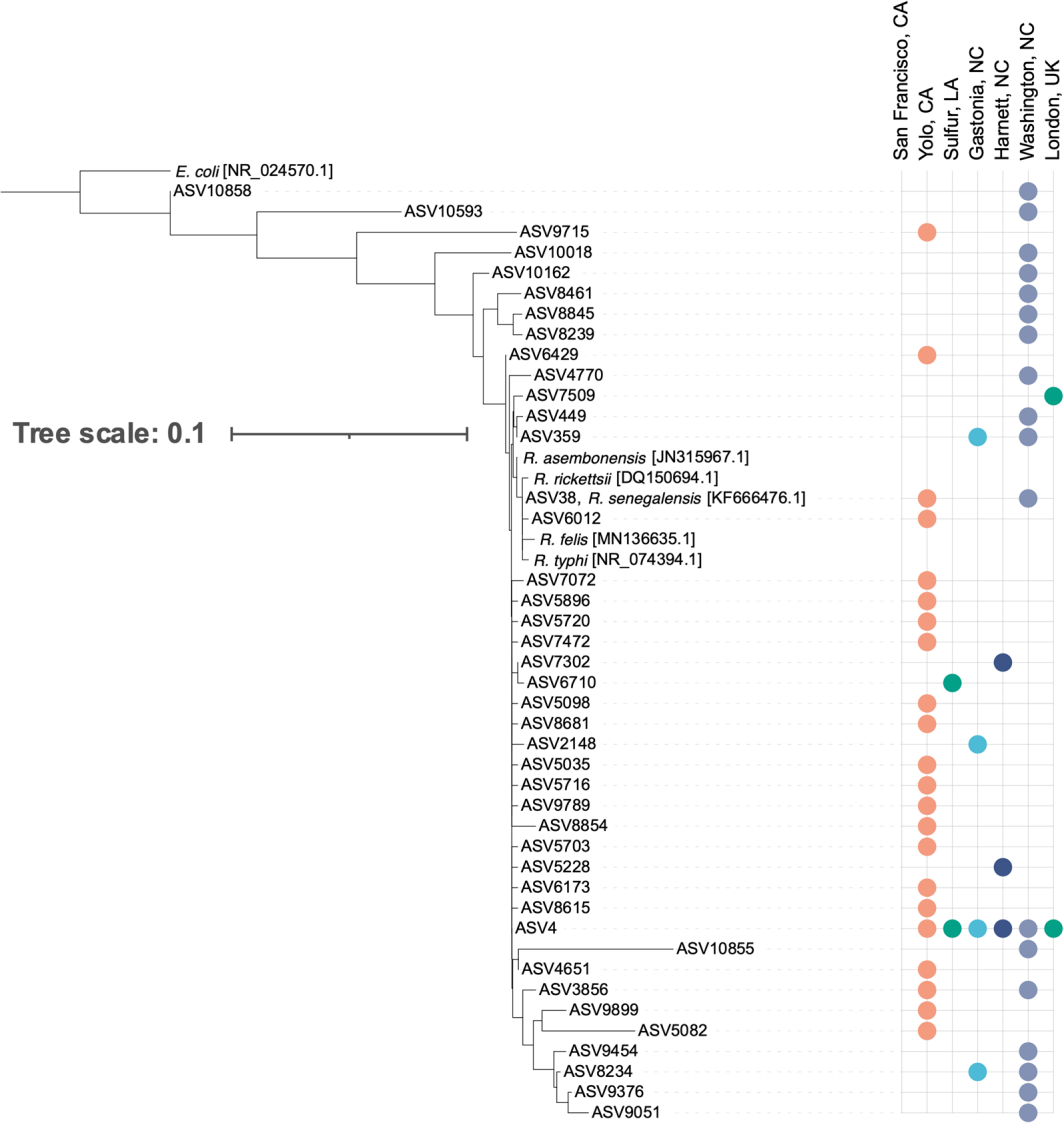
Phylogenetic tree displaying the homology of *Rickettsia* ASVs compared to sequences of known origin with GenBank reference indicated in brackets. Colors in the dot plot indicate the location(s) at which specific ASVs were detected; the locations are labeled on the x-axis

NGS yielded 42 distinct *Rickettsia* ASVs. Read abundance was dominated by a single ASV: ASV4 (99.75% *R. asembonensis*, accession ID JN315973.1) (Fig. 2b). Only one of the 42 ASVs aligned perfectly with a known *Rickettsia*: ASV38 and ‘*Candidatus* Rickettsia senegalensis’ (Fig. 4). No ASV aligned perfectly with *R. felis*. *Rickettsia* ASVs also displayed geographic location-based differences in detection: ASV4 was detected in all locations, ASV38 and ASV3856 were detected in Washington, NC, and Davis, CA, and all other ASVs were found in a single location or two NC locations (Fig. 4).

**Fig. 5:**
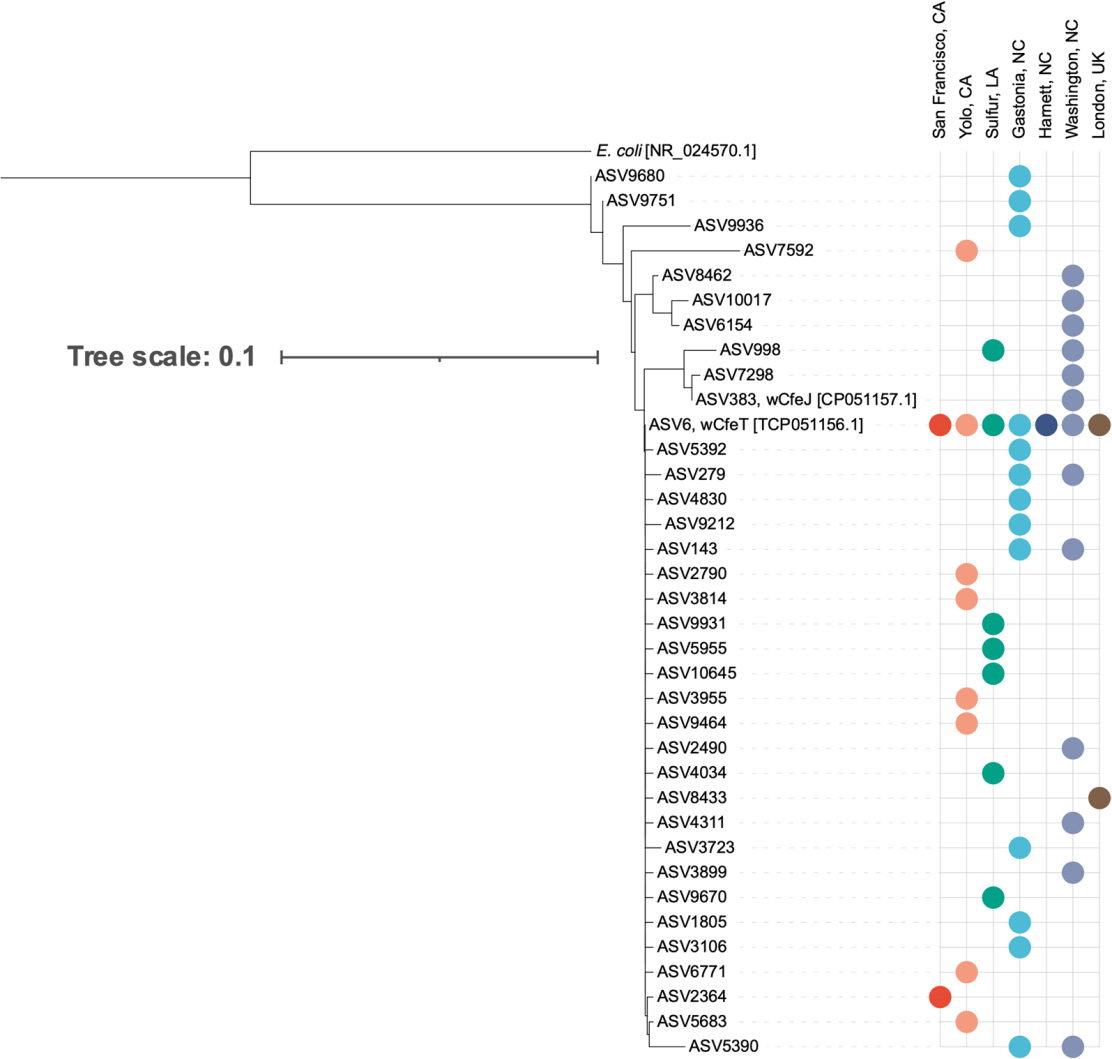
Phylogenetic tree displaying the homology of *Wolbachia* ASVs compared to sequences of known origin with GenBank reference indicated in brackets. Colors in the dot plot indicate the location(s) at which specific ASVs were detected; the locations are labeled on the x-axis

NGS yielded 36 distinct *Wolbachia* spp. ASVs (Fig. 2c). The most abundant *Wolbachia* ASV (ASV6) aligned perfectly with *w*CfeT (accession ID NZ_CP051156.1). ASV383, the fourth most abundant *Wolbachia* ASV, aligned perfectly with *w*CfeJ (accession ID NZ_CP051157.1) [26]. The full diversity of *Wolbachia* ASVs compared to *w*CfeT and *w*CfeJ is shown in Fig. 5. *Wolbachia* ASVs displayed geographic location-based differences in detection: ASV6 was detected in all locations, ASV998 was detected in Washington, NC, and Sulphur, LA, and all other ASVs were found in a single location or two NC locations.

### Flea phylogenetics

**Fig. 6:**
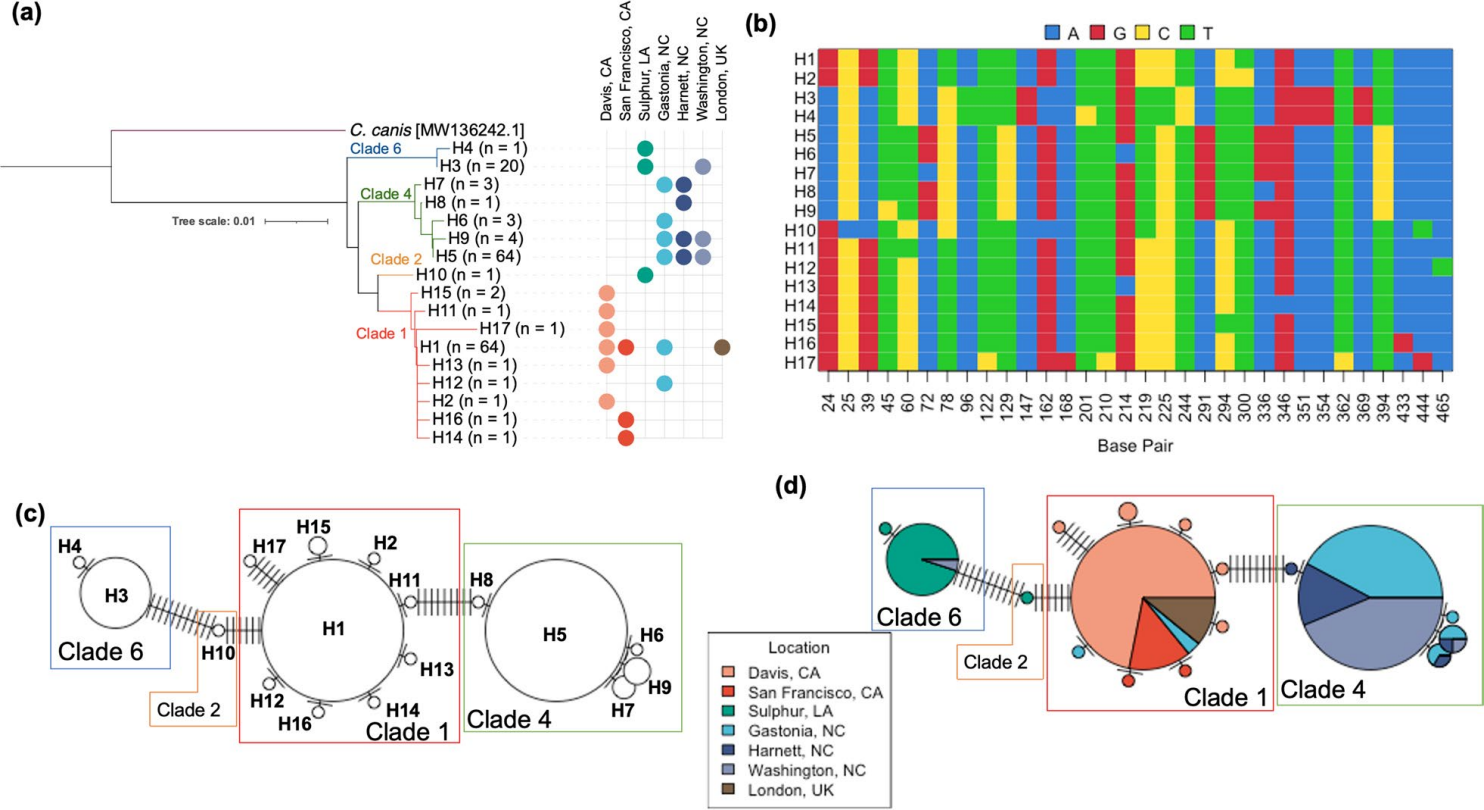
**a** Phylogenetic tree displaying the relationship between identified haplotypes and location at which they were detected. **b** Specific base pair sites identified as sources of variability between haplotypes. **c** Haplotype network displaying the relationship between haplotypes and the **d** location of origin of the fleas from different haplotypes

The *cox1* sequencing identified 17 flea haplotypes with these haplotypes arising from mutations in 32 base pair locations (Fig. 6b). The phylogenetic tree and haplotype network describing the relationship between these haplotypes are in Fig. 6a and c, respectively. A haplotype network displaying the proportion of fleas from each location is shown in Fig. 6d. The α-diversity metric NRI was used to compare the within-group diversity when fleas were grouped by location. NRI indicated that Washington, NC, was the most phylogenetically diverse location among the seven study sites, while London, UK, was the least phylogenetically diverse. To quantify phylogenetic similarity between local flea communities, the β-diversity measure PhyloSor was calculated [52]. This calculation indicated two location-based groups of phylogenetic similarity: one comprised of Sulphur, LA, and the three North Carolina locations and the other comprised of London, UK, and the two California locations.

When comparing these fleas to the haplotypes published by Lawrence et al. [37], our flea haplotypes all fell within previously reported clades: Clade 1 (*n* = 9), Clade 2 (*n* = 1), Clade 4 (*n* = 5), and Clade 6 (*n* = 2), revealing new haplotypes in Clades 1 (*n* = 5), 2 (*n* = 1), 4 (*n* = 3), and 6 (*n* = 1) (Additional file 2: Fig. S2).

**Fig. 7:**
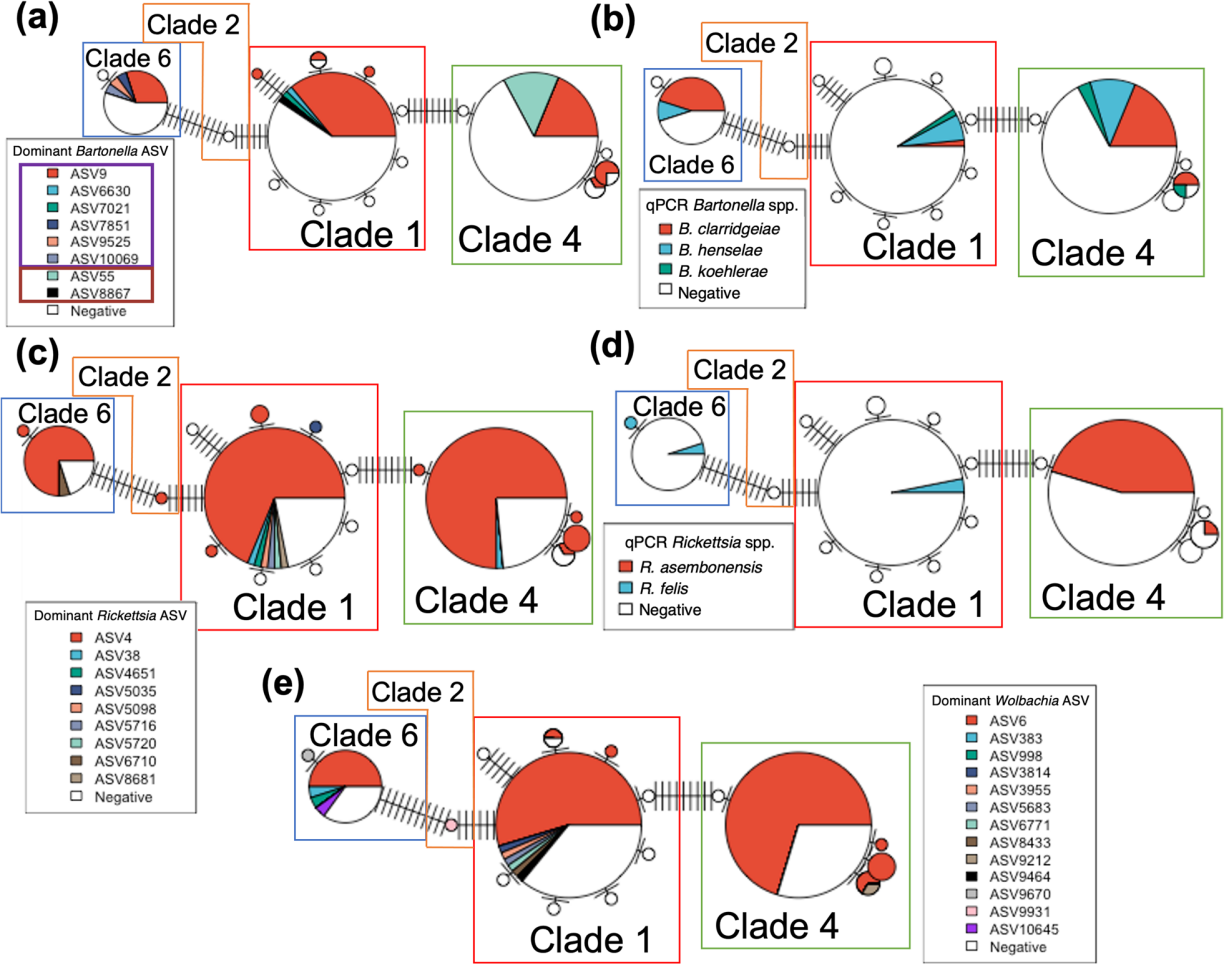
Haplotype network displaying the dominant *Bartonella* ASV from NGS (**a**), *Bartonella* spp. reported by qPCR (**b**), dominant *Rickettsia* ASV from NGS (**c**), *Rickettsia* spp. reported by qPCR (**d**), and dominant *Wolbachia* ASV from NGS (**e**)

Haplotype networks comparing specific flea haplotype to *Bartonella* spp. reported by NGS (dominant ASV) and qPCR sequencing are illustrated in Fig. 7a and b, respectively. Only one minor *Bc*-like ASV was found in multiple fleas (ASV257). These fleas were from two NC locations and belonged to the same haplotype. A single *Bh/Bk-*like ASV was detected in multiple fleas: ASV55. ASV55 was detected in only two haplotypes but four locations. All other fleas containing *Bh/Bk*-like ASVs belonged to other haplotypes, suggesting that *B. henselae* strains may be associated with specific *C. felis* haplotype.

*Wolbachia* ASVs also displayed haplotype-specific occurrence patterns. The dominant *Wolbachia* ASV (ASV6) was detected in all geographic locations and in fleas from three clades (Fig. 7e). Five minor *Wolbachia* ASVs were detected in more than one flea. All of these ASVs were detected in only a single flea clade, with a majority (3/5) found in only one flea haplotype despite four of the *Wolbachia* ASVs occurring in more than one location. ASV998, an ASV detected in Sulphur, LA, and Washington, NC, was detected in two fleas, one from each location. Both of these fleas were assigned to haplotype 3 in clade 6, a haplotype and clade otherwise populated exclusively by Sulphur, LA, fleas. The haplotype network comparing flea haplotype to the dominant *Wolbachia* ASV is illustrated in Fig. 7e.

*Rickettsia* strain prevalence by flea genotype was less clear than that of either *Bartonella* or *Wolbachia*. A dominant *Rickettsia* ASV (ASV4), detected in all locations except San Francisco, CA, was present in fleas from all four clades. Six minor *Rickettsia* ASVs were detected in multiple fleas, all of which were from multiple locations and in fleas from multiple clades. The haplotype network comparing flea haplotype to the dominant *Rickettsia* ASV and qPCR *Rickettsia* spp. is illustrated in Fig. 7c and d, respectively.

### Coinfection

**Fig. 8:**
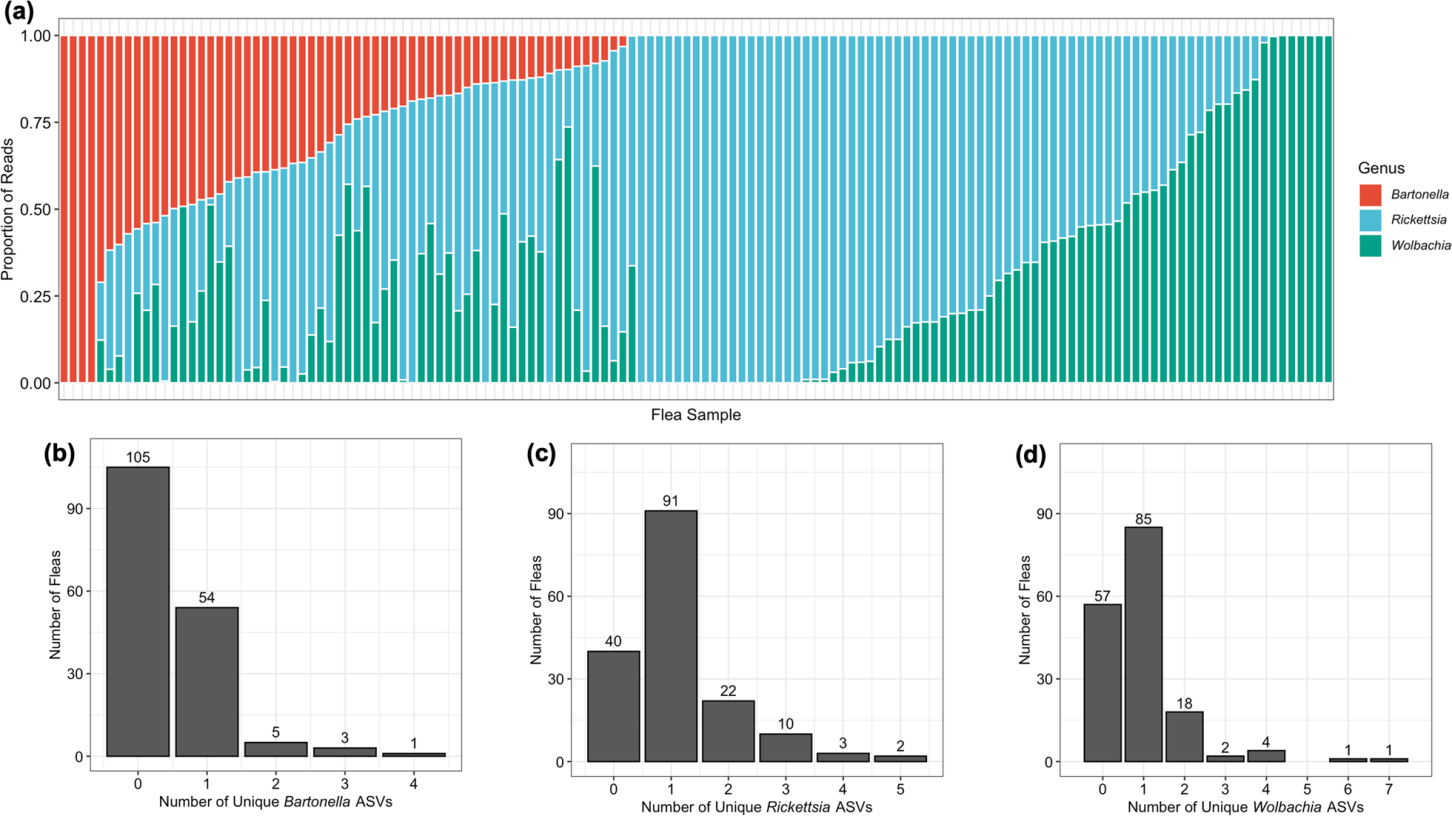
**a** Barchart displaying the proportion of reads from each individual flea assigned to *Bartonella, Rickettsia*, or *Wolbachia* not including other genera. The numbers of fleas with intra-genus coinfection with *Bartonella* (**b**), *Rickettsia* (**c**), or *Wolbachia* (**d**) are indicated in the bottom figures

The majority of fleas in this study were coinfected with more than one of the genera *Bartonella, Rickettsia*, and *Wolbachia* (Fig. 8a). The NGS data also provided support for intra-genus coinfection. Among infected fleas, 14% (9/63) contained more than one *Bartonella* ASV (Fig. 8b), 29% (37/128) had more than one *Rickettsia* ASV (Fig. 8c), and 23% (26/111) had more than one *Wolbachia* ASV (Fig. 8d). It is unlikely that intra-genomic variation between different copies of the 16S rRNA gene can explain these results as *Rickettsia* and *Wolbachia* only have one gene copy and *Bartonella* has two copies, relatively few compared to other bacterial genomes.

### qPCR comparison with NGS

Combining results from both NGS and qPCR diagnostic methods, *Bartonella* spp. sequences were found in 47% (79/168) of fleas. A total of 63 fleas were identified as infected with *Bartonella* spp. by NGS compared to 41 fleas by qPCR. When qPCR is considered the reference standard, NGS had an overall sensitivity of 61% and specificity of 70% for detection of *Bartonella* spp. in fleas (Table 3). The proportion of qPCR-positive fleas for which NGS also returned a positive detection did not vary by the qPCR species, NGS ASV, or qPCR primer set.

**Table 3:** Proportion of fleas from which *Bartonella* spp. was detected by qPCR and NGS

	NGS positive	NGS negative
qPCR positive	25	16
qPCR negative	38	89

Despite NGS detecting *Rickettsia* ASV(s) in 76% (128/168) fleas, we were only able to sequence clean *Rickettsia* spp. amplicons from 20% (34/168) of fleas by qPCR. When qPCR is considered the reference standard, NGS had an overall sensitivity of 94% and specificity of 28% for detection of *Rickettsia* spp. in fleas (Table 4). The fraction of qPCR-positive fleas for which NGS also returned a positive detection did not vary by the qPCR species, NGS ASV, or qPCR primer set.

**Table 4:** Proportion of fleas from which *Rickettsia* spp. was detected by qPCR and NGS

	NGS positive	NGS negative
qPCR positive	32	2
qPCR negative	96	38

## Discussion

This study used 16S NGS to analyze the eubacterial microbiome of *C. felis* fleas removed from free-roaming cats across diverse geographic locations. As expected, we documented widespread infection with known *C. felis* microbiome members (*Bartonella, Rickettsia*, and *Wolbachia)*. Comparisons between negative controls and flea samples combined with literature search facilitated the selection of *Achromobacter, Peptoniphilus*, and *Rhodococcus* as candidate *C. felis* microbiome members. Multiple *Bartonella, Rickettsia*, and/or *Wolbachia* ASVs were amplified from individual fleas, suggesting that coinfection both between these genera and among species/strains within each of these genera is a common occurrence. Almost all minor ASVs were detected in fleas from only a single location or in multiple locations from the same state suggesting location-based strain variation. *Ctenocephalides felis* phylogenetic clade and haplotype were associated with geographic location. Combined *C. felis* phylogenetic and NGS data more strongly supported a relationship between flea phylogeny and infecting *Bartonella* and *Wolbachia* ASV than flea phylogeny and *Rickettsia* ASV.

NGS paired with robust data analysis is a promising approach for identifying true microbiome members in low-biomass or contaminant prone samples. It is critical that stringent controls and comprehensive techniques, such as those used in this study, are utilized to identify vector microbiome members. We did not detect *Elizabethkingia* or *Snodgrassella* spp. DNA in this study, as previously reported in a study by Vasconcelos et al. [3]. None of the genera reported by Cohen et al. in *Synosternus cleopatrae* fleas, other than those noted in our analyses, were deemed microbiome members due to either their absence in flea samples or their presence in negative controls [86]. Our inability to either detect or recognize previously reported genera as *C. felis* microbiome members may be due to these genera not being present in the fleas in our study, or because those genera are unrecognized contamination.

Our finding that most minor ASVs from the three known *C. felis* genera (*Bartonella, Rickettsia*, and *Wolbachia)* were only detected in a single or geographically close location(s) may imply that diversity within these bacterial genera is associated with geographical location. The association of bacterial diversity with *C. felis* genotype remains to be elucidated. The full implications of these findings are unknown; however, specific strains of *Bartonella* spp. are known to exhibit highly varying pathogenicity [87] and display differences in factors relating to the risk and severity of infection. Studies that further characterize diversity within *C. felis*-associated bacteria are necessary, as the relatively short (402 bp) sequence of the 16S gene amplified by NGS is not designed to resolve strain-based variability in virulence factors or pathogenic potential.

Regarding flea phylogeny, our data reemphasize how limited our knowledge is of *C. felis* diversity in the US as a sample of 168 fleas identified seven new haplotypes within the previously reported clades [37]. Comparing our flea haplotypes to those reported by Lawrence et al., a majority of the California fleas were assigned to Clade 1, a temperate clade, while Sulphur, LA, fleas were assigned primarily to Clade 6, a tropical II clade and a single flea in Clade 2, a temperate clade. North Carolina fleas were primarily assigned to Clade 4, a tropical I clade, with a limited number of fleas in Clade 1 or Clade 6.

Although not the first study to report both flea genotype and pathogen carriage status [2, 20], this study reports a large sample of *C. felis* genotype and *Bartonella, Rickettsia*, and *Wolbachia* infection status. Our data provide evidence that *C. felis* genotypic haplotype is most likely to be related to the infecting *Bartonella* and *Wolbachia* strain but less likely to be related to *Rickettsia* strain. The dominant *Bh/Bk*-like ASV was detected in only two haplotypes despite being present in four locations. Furthermore, no two *Bh/Bk*-like ASVs infected the same flea haplotype. The single minor *Bc*-like ASV detected in multiple fleas was detected in multiple geographic locations, but only one flea haplotype. For *Wolbachia* spp., we determined that the single Washington, NC, flea assigned to clade 6 was infected with *Wolbachia* ASV998, a clade and ASV only found to be inhabited by or infecting Sulphur, LA, fleas of clade 6. A majority (3/5) of all minor *Wolbachia* ASVs were detected in only a single clade. These findings indicate that *C. felis*-associated bacteria may display previously unidentified vector genotype by pathogen genotype relationships or divergent evolution with the fleas they inhabit. Further study is necessary to establish the relationship between geographic location, flea haplotype, microbiome diversity, pathogen prevalence, and strain delineation using markers with higher resolution than the short-read 16S target utilized.

Our analyses also allowed us to compare detection of *Bartonella* spp. in NGS versus qPCR. Given the lack of a true gold standard, we utilized qPCR, which is not a 100% sensitive *Bartonella* diagnostic assay [88], a factor that likely contributed to NGS having a specificity of only 68% (89/127). It is likely that the additional 38 fleas positive by NGS were truly infected at a level below qPCR detection. NGS did not detect *Bartonella* ASVs in 39% (16/41) of the fleas positive by qPCR and sequencing. Our NGS findings indicated that *B. clarridgeiae* is the most common *Bartonella* spp. in *C. felis*, a conclusion that was supported by qPCR findings and previous literature [89, 90]. Comparing the agreement between NGS and qPCR, which was only 68% (114/168), it is likely that the sensitivity of both methods is suboptimal.

The dominant *Rickettsia* ASV (ASV4) detected in our analysis is most closely related to *R. asembonensis*, not *R. felis*, suggesting that *C. felis* in the US may more frequently harbor *Rickettsia* spp. other than *R. felis*. Beyond the dominant ASV, we found a very diverse collection of *Rickettsia* ASVs that have not been characterized. These findings reinforce the importance of exploring the epidemiology of flea-associated *Rickettsia* spp. in future surveys [7, 91]. In the absence of a gold standard assay, we again compared detection in qPCR and NGS and found that NGS reported a sensitivity of 94% (32/34) for the detection of *Rickettsia* spp. Unfortunately, the 28% (38/134) specificity indicates a failure of the NGS or qPCR assay. Due to the large diversity of *Rickettsia* ASVs detected by NGS, we suspect that the qPCR assays lacked specificity for the larger diversity of *Rickettsia* present in these fleas. Based on NGS results, *Rickettsia* co-infection data suggest that contrary to the patterns observed in ticks [32], colonization with one *Rickettsia* does not inhibit colonization with a second *Rickettsia* spp. in *C. felis*.

We detected *Wolbachia* ASVs in 66% (111/168) of fleas, substantially more than the 21% previously reported by qPCR [22]. The high proportion of fleas with coinfecting *Wolbachia* ASVs is on par with previous evidence of *Wolbachia* co-infection in laboratory flea colonies. Dricoll et al. proposed a selection for *w*CfeT over *w*CfeJ in nature, which agrees with our finding that ASV6 (100% *w*CfeT) was the dominant *Wolbachia* ASV and ASV383 (100% *w*CfeJ) was observed in only a single flea [26].

An important limitation of the present study was the low biomass of each *C. felis* sample, which likely allowed contaminant DNA from extraction kits and cross-contamination to dominate data [28]. The impact of this was mitigated by use of decontam [45] and literature search of proposed microbiome members; however, it restricted our ability to perform whole microbial community analysis. Additional limitations included our sampling of *C. felis* from free-roaming cats, which may not accurately represent the fleas from client-owned cats. Sex was not determined for the sampled *C. felis*, preventing comparison between male and female bacterial carriage [86]. *Rickettsia felis* prevalence may be underestimated in this study as the cat is not considered a reservoir host [12]. Instead, current literature implicates dogs, rodents, opossums, or fleas themselves as reservoir hosts for *R. felis* [92]. Concerning diagnostic techniques, qPCR, while specific as results were confirmed by DNA sequencing, suffers from a lack of sensitivity and therefore likely underestimated pathogen prevalence [88].

## Conclusions

The present study reports diverse *Bartonella, Rickettsia*, and *Wolbachia* spp. colonizing the *C. felis* microbiome, which varies by *C. felis* geographic origin and haplotype. While the read abundance of each of these genera was clearly dominated by a single ASV, multiple other ASVs were also identified, likely representing previously uncharacterized strains or species with unknown pathogenicity. Fleas were found to be phylogenetically diverse with haplotype diversity varying by location of origin and multiple new flea haplotypes detected. This work reinforces the importance of future research investigating the diversity of *C. felis* and their pathogenic and non-pathogenic microbiome members to guide diagnosis, treatment, and flea-borne pathogen control recommendations.

## Supplementary Information

**Additional file 1: Figure S1.** Decontam assigned metric score for ASVs from the genera identified to have at least one ASV with a decontam score above the minimum from *Bartonella, Rickettsia *and *Wolbachia *(> 0.89). Those identified as candidate microbiome members on the basis of literature search are outlined in green while the known microbiome members are outlined in red.

**Additional file 2: Figure S2. **Phylogenetic tree aligning haplotypes detected in the present study to those reported by Lawrence et al.^37^ Nodes which contained haplotypes reported by this study are highlighted in red and indicated by a capital H.

## Data Availability

The datasets supporting the conclusions of this article are available in a Dryad repository (https://doi.org/10.5061/dryad.3ffbg79km).

## Abbreviations

ASV: Amplicon sequence variant
FRDC: Free-roaming domestic cat
iTOL: Interactive tree of life
NGS: Next generation sequencing
NRI: Net relatedness index
qPCR: Quantitative polymerase chain reaction
TNR: Trap-neuter-release
