# Supplementary figures and images for: Identification of microbial taxa present in Ctenocephalides felis (cat flea) reveals widespread co-infection and associations with vector phylogeny
Source: Parasit Vectors. 2022 Oct 31;15:398. doi: 10.1186/s13071-022-05487-1 (PMC9623975; doi:10.1186/s13071-022-05487-1)

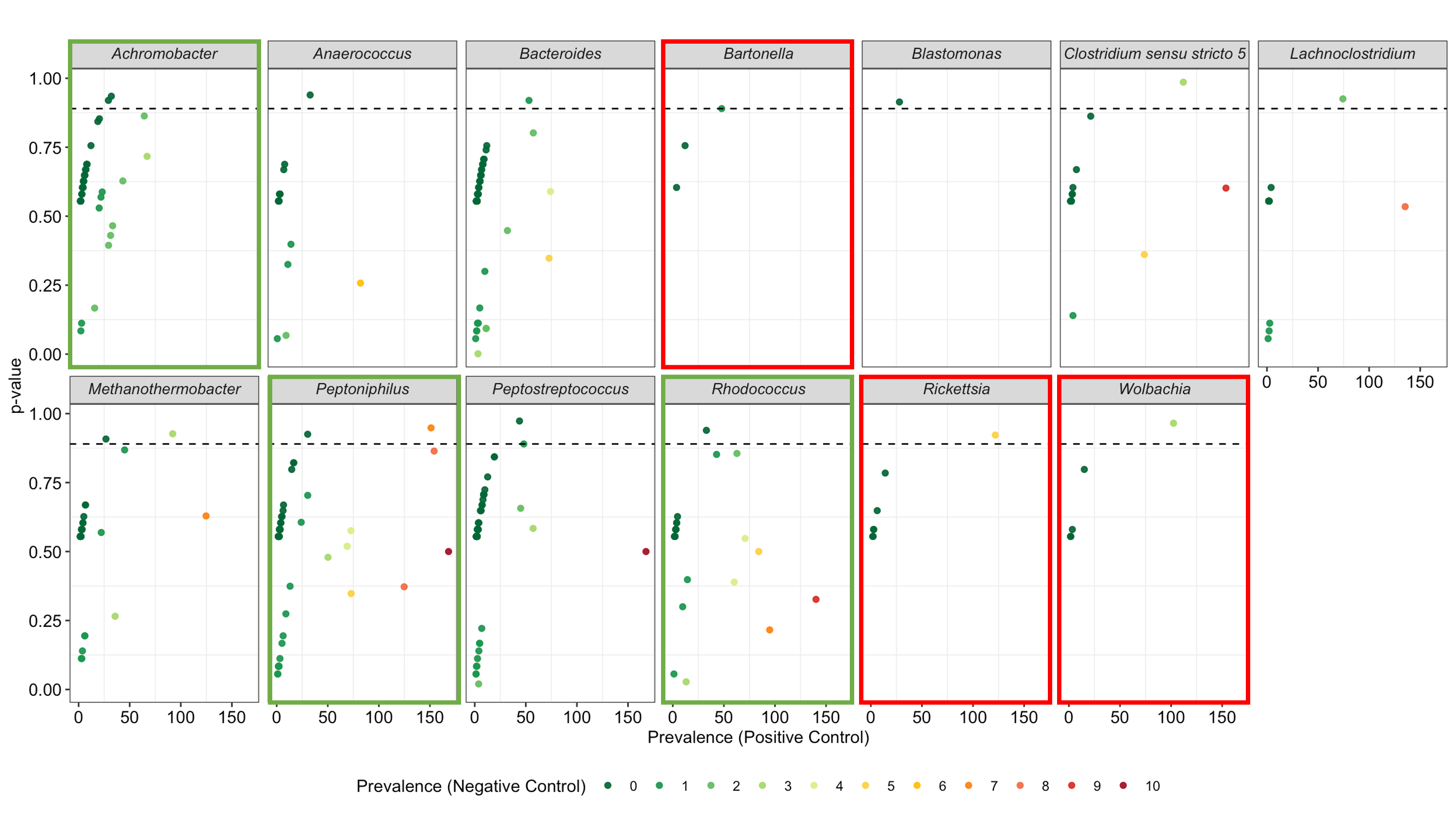

Supplement: Supplementary file 1 — Additional file 1: Figure S1. Decontam assigned metric score for ASVs from the genera identified to have at least one ASV with a decontam score above the minimum from Bartonella, Rickettsia and Wolbachia (> 0.89). Those identified as candidate microbiome members on the basis of literature search are outlined in green while the known microbiome members are outlined in red. [file 13071_2022_5487_MOESM1_ESM.tiff]

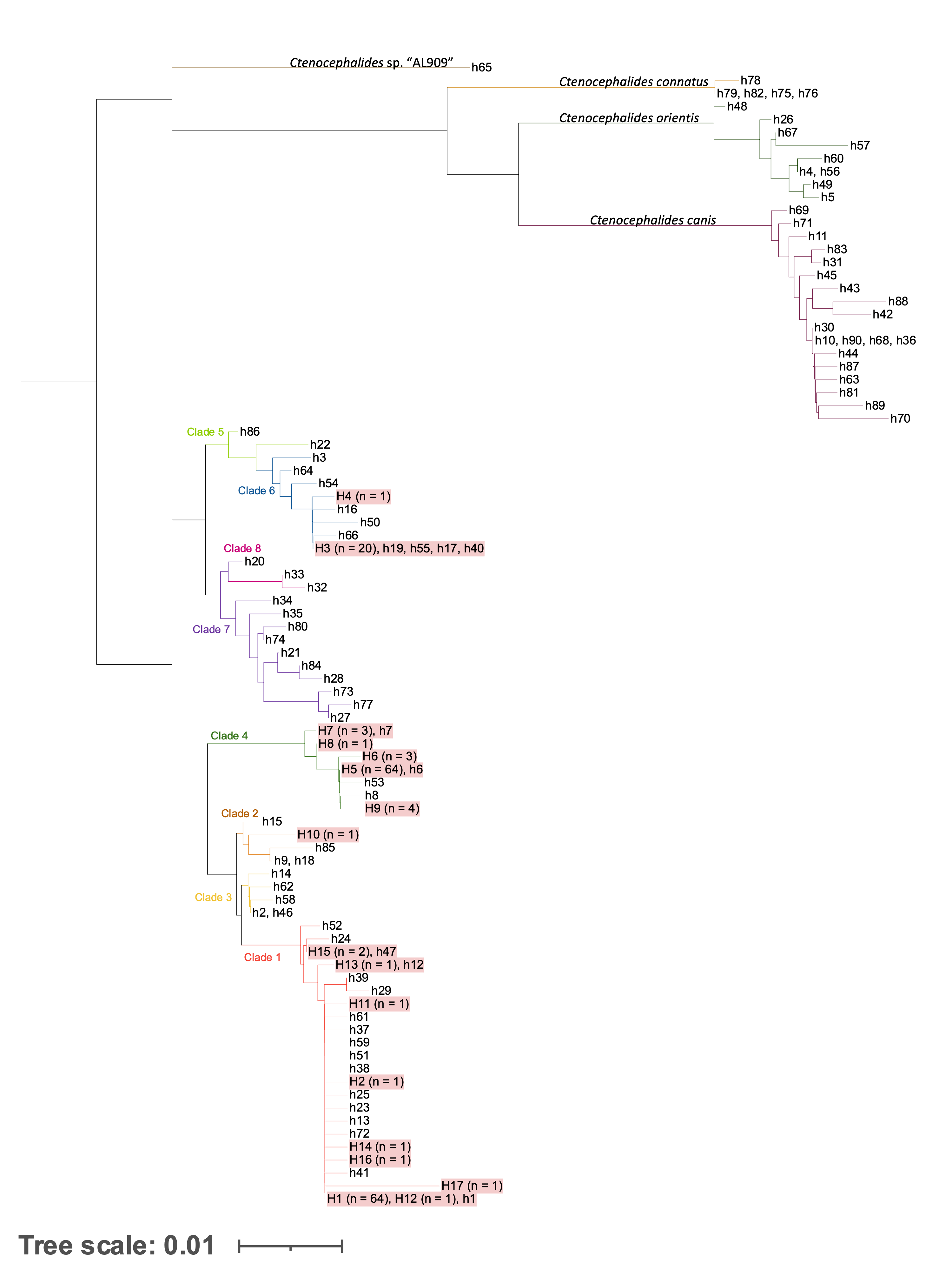

Supplement: Supplementary file 2 — Additional file 2: Figure S2. Phylogenetic tree aligning haplotypes detected in the present study to those reported by Lawrence et al.37 Nodes which contained haplotypes reported by this study are highlighted in red and indicated by a capital H. [file 13071_2022_5487_MOESM2_ESM.tiff]
